# Acceptability of injectable pre-exposure prophylaxis among people who inject drugs in three urban U.S. settings

**DOI:** 10.1186/s12879-022-07572-3

**Published:** 2022-09-14

**Authors:** Adrian R. King, Saanchi Shah, Laura A. Randall, Paula M. Frew, Anne Spaulding, HBOU Study Team, Ian W. Holloway

**Affiliations:** 1 UNLV School of Public Health and UNLV Population Health and Health Equity Initiative, University of Nevada Las Vegas, Las Vegas, NV USA; 2 Department of Community Health Sciences, UCLA Fielding School of Public Health, Los Angeles, CA USA; 3 Department of Epidemiology, Rollins School of Public Health, Emory University, Atlanta, USA; 4 Department of Social Welfare, UCLA Luskin School of Public Affairs, Los Angeles, CA USA; 5 Present Address: ICF, 3 Corporate Square NE, Building 3, Suite 370, Atlanta, GA 30329 USA; 6 Present Address: Merck & Co., Inc., Kenilworth, NJ USA

**Keywords:** Pre-exposure prophylaxis, PrEP, PWID, Injection drug use, HIV prevention

## Abstract

**Background:**

Outbreaks of new HIV transmission among people who inject drugs (PWID) are a major public health concern. Oral daily PrEP, has been identified as a critical addition to the biomedical toolkit for this population. However, limited research on the acceptability of long-acting injectable PrEP has been conducted with this population.

**Methods:**

We conducted a cross sectional multi-site survey with 1127 participants from May 2019–February 2020 to assess the acceptability of novel PrEP regimens. We computed bivariate and multivariable logistic regressions to evaluate correlates of the outcome variable: acceptability of 3-month injectable-PrEP. SAS v.9.4 was used to conduct statistical analysis.

**Results:**

Limited knowledge of or use of PrEP, past or present, was evident within the sample. Injection drug use in the past six months was significantly associated with LA injectable PrEP acceptability, with the odds of acceptability being 1.885 (CI: 1.376, 2.582) times greater than those who did not inject drugs. After adjusting for confounders, injection drug use was significantly associated with the outcome, such that the odds of acceptability of LA injectable PrEP were 1.705 (CI: 1.198, 2.427) times greater among PWID compared to those who did not inject drugs (*p* < 0.03). The results demonstrate acceptability (38.2%) in a durable (3-month) injectable PrEP modality among participants who also identified as PWID.

**Conclusions:**

PrEP promotion efforts among PWID to increase access to long-acting injectable PrEP are necessary. Through efforts to increase acceptance and regular use of long-acting injectable PrEP, public health strategies may be able to effectively lessen chances of future HIV outbreaks among PWID.

## Background

Substance misuse, spurred by the opioid epidemic, has resulted in a number of public health crises related to opioid overdose and increased Human Immunodeficiency Virus (HIV) incidence among people who inject drugs (PWID). In the United States (US), injection drug use has affected over 6.5 million individuals, and estimates of current PWID top 750,000 persons across the US [1]. Recent research indicates that some 20% of non-institutionalized adults and adolescents reported recent illicit drug misuse (e.g., marijuana, prescription pain relievers, opioids, heroin) [2]. Research also shows that in 2018 alone, over 10 million Americans misused opioids and a significant portion of opioid misuse co-occurred with heroin use [2]. More recently, overdoses caused by opiates and injection drug use have increased exponentially, in large part due to the COVID-19 pandemic. In 2019 over 70,000 drug overdoses were reported in the US [3]. In 2020, drug overdoses increased by 29.4%, according to preliminary data, resulting in over 93,000 deaths, nearly 70,000 involving opioids [4].

Behaviors increasing PWID’s risk of HIV transmission include sharing of needles with other injection drug users (IDU), reuse of one’s own needles, and improper cleaning of needles (e.g., using bleach) [5]. Even without the risk of sharing needles or syringes, considerable HIV transmission risk exists through sharing of injection drug preparation equipment (IDPE) (e.g., cookers and filters) as PWID often do not consider this risk [5]. Additionally, PWID who are living with HIV are often unaware of their HIV status and may unknowingly transmit HIV to others [6]. Data from the Centers for Disease Control and Prevention (CDC) indicate that nearly 10% of new HIV diagnoses in 2017 were attributed to injection drug use; of these, males and persons between ages 25–44 years experienced a higher burden of new HIV transmission [7]. HIV transmission through sexual behavior is also common amongst PWID, especially among younger males [8].

Pre-exposure prophylaxis (PrEP) is a biomedical strategy for HIV prevention currently approved for use in the U.S. using daily oral emtricitabine and tenofovir alafenamide (or tenofovir disoproxil fumarate – Emtricitabine + TAF/TDF) [9, 10]. In 2015, the World Health Organization (WHO) explicitly stated that any person at a substantial risk for HIV acquisition should be offered Emtricitabine + TAF/TDF in an effort to prevent future HIV transmission [11]. Research has identified the need for development and testing of novel PrEP delivery modalities to alleviate various barriers to access [12]. The need for additional research on novel PrEP modalities has continued, and some public health entities have supported a 2-1-1 (or event driven) tenofovir plus emtricitabine dosing strategy for men who have sex with men (MSM) [13].

Recent innovation has also identified injectable PrEP as a potential modality to increase PrEP acceptability and usage, and lessen potential barriers to PrEP uptake and adherence. Research has shown that long-acting (LA) injectable PrEP is acceptable among a number of populations and may reduce barriers to uptake [14–17]. Research has also shown considerable acceptability of daily oral PrEP among PWID, although additional data is warranted [18–22]. Additionally, research has shown LA injectable PrEP to be safe and it has been approved for use among at risk adults to prevent sexually-acquired HIV by the U.S. Food and Drug Administration as of December 2021 [23].

Despite broad recommendations and uptake in other populations, scant research has focused on PrEP use among PWID. Research from 2018 identified that while some PWID believe that PrEP would be beneficial and acceptable within their communities, there is limited PrEP-specific knowledge in this population [18, 19]. Use and acceptability of PrEP among PWID also depends heavily on optimizing PrEP regimens and technologies given situational barriers like transportation, cost, insurance coverage, and access to healthcare services [12, 24]. While research on novel PrEP modalities (e.g., injectable) to address some of these barriers has identified acceptability of new methods, it was conducted among sexual minority men and did not focus on PWID [22]. Further, findings suggest general acceptability and efficacy of oral Emtricitabine + TAF/TDF among PWID [20, 25, 26]; however, research on acceptability of injectable PrEP with this population is still limited [27, 28]. Our study aimed to identify the acceptability of LA injectable PrEP among PWID. Developing an understanding of acceptability is critical to ending the HIV epidemic, as this information may assist public health programs to effectively create HIV prevention interventions to increase PrEP coverage among PWID once LA injectable PrEP becomes more widely available.

## Methods

We conducted a community-engaged research project in collaboration with syringe service programs (SSPs) in three urban US settings (Las Vegas, Los Angeles, and Atlanta) from May 2019 to February 2020. Our project included the delivery of an online tablet-based questionnaire which participants completed independently while seeking services at participating SSP locations. SSP location staff were involved in our recruitment efforts and facilitated relationship building with this population as our project staff did not have this established relationship. Data collection sites were chosen based on characteristics of the population of people using opioids, particularly those who may be vulnerable to HIV. The selected cities were Atlanta, Georgia; Los Angeles, California; and Las Vegas, Nevada. SSP’s were included in this project as project team leadership had previously developed collaborative partnerships with these entities through long-standing relationships between project leadership and SSP staff.

### Sampling

Eligible participants included those who had accessed designated SSP’s services, reported using opioids in the previous six months, were 18–69 years of age, had English or Spanish language comprehension, and provided voluntarily consent to participate. A sampling target of 400 per city (total of 1200) was chosen to fit within funding restraints while providing an adequate sample size for city-specific and comparative analyses. This sampling target also ensured adequate samples of subpopulations such as LGBTQ + people. For completion of the questionnaire respondents were provided a nominal well-being item (e.g., hand sanitizer, hygiene kit, sunscreen, $5 McDonalds giftcard).

### Study instrument

Survey data were collected using an interview administred, tablet-based online questionnaire, which enrolled participants completed on electronic tablets at SSPs. Our survey was developed with valid and reliable measures and was offered to participants in English and Spanish. A full explanation of the survey and protocol for data collection have been previously published [29].

Our outcome of interest was self-reported acceptability of 3-month LA injectable PrEP. Participants were asked the following question regarding LA injectable PrEP: “Please indicate how likely you would be to use injectable PrEP that could be taken every three months” with the following response categories: ‘Not very likely’, ‘Somewhat unlikely’, ‘Neither likely nor unlikely’, ‘Likely’ and ‘Extremely likely’. Response categories were collapsed such that ‘likely’ and ‘extremely likely’ were recoded as ‘likely’ while the remaining groups were recoded as ‘not likely’. We also asked participants about acceptability of one-month LA injectable PrEP, although this was not our primary outcome of interest.

We used the following independent variables: injection drug use, age, race/ethnicity, gender identity, sexual orientation, location of participants, U.S nativity, educational attainment, employment status, controlled environment, access to insurance, annual household income, SNAP benefits, and unstable housing/homelessness.

### Substance use

Injection drug use in the past 6 months was the main independent variable. This was recoded by combining affirmative responses to injection (intravenous [IV] vs. non-IV) as the route of administration for any substances. We also captured data on other substance usage through questions which asked if participants had used substances (e.g., heroin, cannabis, opioids, amphetamines) in the past six months.

### Race/ethnicity

Both race and ethnicity were self-reported by participants. The two variables, race and ethnicity, were recoded into a single variable so that Hispanic, non-Hispanic White, Non-Hispanic Black, and Other were the subsequent categories of this newly collapsed variable.

### Educational attainment

The original eight response categories for highest educational attainment were recoded into three new categories: ‘Up to High School’; ‘High School to Associate’s’; and ‘Higher education’.

### Relationship status

Response categories were recoded to reflect ‘Never married’, ‘Ever married’ and ‘Partnered’.

### Employment status

Categories to describe employment status were coded as follows: ‘Unemployed and looking for work,Unemployed and not looking for work, Employed, and Out of Workforce.

### Controlled environment

Responses to questions about living in a controlled environment in the last six months (i.e., jail, prison, alcohol or drug treatment center, psychiatric treatment center) were recoded as categorized as Yes, No, and Never.

### Access to insurance

Participants who reported any access to insurance were recoded as ‘Yes’. Participants without reported access to insurance were recoded into ‘No access’, while those who were unsure about their insurance access were recoded as ‘Unsure’.

### Gender identity/sexual orientation

Using the variables gender identity and sexual orientation, a new variable was constructed with four categories to understand differences between cisgender heterosexual men, cisgender heterosexual women, cisgender sexual minority men and cisgender sexual minority women. Participants not identifying as either ‘male’ or ‘female’ were not included in analysis, due to small sample size (n = 2).

### Pre-exposure prophylaxis (PrEP)

Respondents provided answers to a series of questions pertaining to PrEP awareness, knowledge, acceptability, and use. Respondents were provided a brief description of what PrEP is and that it can “lower their risk of getting HIV”. Data derived from these responses were not recoded and data are presented with the original answer options. Questions asking about likelihood to use novel PrEP methodologies (e.g., 1-month injectable, 3-month injectable, lubricants, spermicides) utilizing a likert scale of likelihood with ‘Not Very Likely’ (1) and ‘Extremely Likely’ (5) on either end of the spectrum. Our instrument also asked participants if they had ever heard of or used PrEP and whether they were currently using PrEP. We also asked participants if they knew where they could get PrEP if they wished to start taking PrEP.

### Data storage

Participants were given an identification number. We obtained demographic information during the screener and survey, which was collected on a Health Insurance Portability and Accountability Act (HIPAA)-compliant survey administration platform, Qualtrics, (Provo, UT). Qualtrics safeguards all data and uses secure data centers to ensure the highest protection per HITECH requirements. Previous publications have detailed our protocol, including participant inclusion criteria and sampling, field activities including informed consent, and analytic strategy [25]. Our project is among the first to capture a holistic view of health behaviors and to examine PrEP knowledge and LA injectable PrEP acceptability among PWID in these geographies.

### Data analysis

SAS v.9.4 was used to conduct statistical analysis. In total, 1127 individuals consented and completed the survey. Fifty individuals who reported living with HIV were not included in the analysis. Among the 1077 individuals who responded to questions pertaining to PrEP, 80 individuals did not respond to the question regarding 3-month injectable-PrEP acceptability and therefore, were not included in the final analytic sample, which was comprised 997 participants across the three sites. We computed bivariate and multivariable logistic regressions to evaluate correlates of the outcome variable: acceptability of 3-month injectable-PrEP.

Based on contextual knowledge and bivariate analyses, a set of candidate variables were introduced for model specification to evaluate factors contributing to acceptability of LA injectable PrEP. A stepwise strategy was used to select the most parsimonious model. This model was chosen because it had minimum AIC compared to models with fewer variables.

## Results

Full participant demographics stratified by acceptability of LA injectable PrEP are described in Table 1. Almost one third (n = 322; 32.4%) indicated that they were likely to accept monthly injectable PrEP. Of those likely to accept LA (3-month) injectable PrEP (n = 381; 38.2%), the majority reported being between 26–45 years old (n = 224; 58.8%), white (n = 201; 54.0%), male (n = 211; 55.4%), having received up to a high school education (n = 187; 49.5%), and having less than $20,000 in annual income (n = 269; 70.8%). Over half (n = 217; 57.0%) identified as currently homeless. Recent substance use (last 30 days) among this group varied with most (n = 307; 80.6%) reporting heroin use, over half (n = 224; 58.8%) reporting amphetamine use, over half (n = 205; 53.8%) reporting cannabis use, and over one third (n = 153; 40.2%) reporting recent opiate use. Most (n = 313; 82.2%) also self-reported injection substance use within the last 6 months.

**Table 1:** Characteristics of individuals (not positive for HIV) who are likely or unlikely to accept injectable PrEP every 3 months in the Health Behaviors among Opioid Users study (N = 997)

	3-month injectable PrEP acceptability	
Characteristic	Likely (N = 381)	Not likely (N = 616)
	N (%)	N (%)
Total	381 (38.2)	616 (61.8)
Age groups		
18–25	24 (6.30)	44 (7.14)
26–35	128 (33.60)	195 (31.66)
36–45	96 (25.20)	156 (25.32)
46–55	88 (23.10)	128 (20.78)
56–65	42 (11.02)	81 (13.15)
66 or older	3 (0.79)	12 (1.95)
Race/ethnicity		
Hispanic	73 (19.6)	163 (27.6)
White	201 (54)	247 (41.9)
Black	74 (19.9)	135 (22.9)
Other	24 (6.5)	45(7.6)
Gender identity		
Male	211 (55.38)	402 (65.26)
Female	166 (43.57)	213 (34.58)
Other	2 (0.52)	0
Decline to answer	2 (0.52)	1 (0.16)
Gender/sexual orientation		
Heterosexual men	175 (46.9)	358 (59.1)
Heterosexual women	104 (27.9)	140 (23.1)
Sexual minority men	35 (9.4)	38 (6.3)
Sexual minority women	59 (15.8)	70 (11.6)
Location		
Las Vegas	157 (41.21)	231 (37.5)
Los Angeles	131 (34.38)	265 (43.02)
Atlanta	93 (24.41)	120 (19.48)
Born in the US		
Yes	362 (95.01)	582 (94.48)
No	15 (3.94)	32 (5.19)
Not sure	1 (0.26)	1 (0.16)
Decline to answer	3 (0.79)	1 (0.16)
Education		
Up to high school	187 (49.47)	387 (63.34)
Some college/vocational/associate degree	160 (42.33)	188 (30.77)
Bachelor's/Master's/Doctorate degree	31 (8.20)	36 (5.89)
Missing	8	
Relationship status		
Never married	196 (52.13)	325 (53.72)
Ever married	140 (37.23)	216 (35.70)
Partnered	40 (10.64)	64 (10.58)
Missing	16	
Employment status		
Employed	83 (22.19)	124 (20.5)
Unemployed and looking for work	139 (37.17)	230 (38.02)
Unemployed and not looking for work	60 (16.04)	99 (16.36)
Out of workforce	85 (22.73)	139 (22.98)
Other	7 (1.87)	13 (2.15)
Missing	18	
Controlled environment in the last 6 months		
Yes, in the last 6 months	150 (39.79)	256 (42.31)
Not held in a controlled environment in the last 6 months	165 (43.77)	228 (37.69)
Never held in a controlled environment	43 (11.41)	68 (11.24)
Decline to answer	19 (5.04)	53 (8.76)
Missing	15	
Insurance access		
Yes	262 (68.77)	423 (68.67)
No	100 (26.25)	138 (22.4)
Unsure	19 (4.99)	55 (8.93)
Missing	9	
Annual household income		
Less than $20,000	269 (70.79)	422 (69.07)
$20,001–$40,000	37 (9.74)	60 (9.82)
$40,001–$60,000	16 (4.21)	25 (4.09)
$60,001–$80,000	7 (1.84)	10 (1.64)
$80,001–$100,000	5 (1.32)	3 (0.49)
$100,001–$120,000	0	4 (0.65)
$120,001–$140,000	3 (0.79)	1 (0.16)
$140,001–$160,000	0	3 (0.79)
$160,001–$180,000	0	1 (0.16)
$180,001–$200,000	0	2 (0.33)
$200,001 or more	5 (1.32)	8 (1.31)
Decline to answer	38 (10)	72 (11.78)
Missing	6	
SNAP benefits		
Yes	220 (58.05)	345 (56.74)
No	146 (38.52)	236 (38.82)
Don't know	5 (1.32)	18 (2.96)
Decline to answer	8 (2.11)	9 (1.48)
Missing	10	
Currently homeless		
Yes	217 (56.96)	378 (62.27)
No	145 (38.06)	204 (33.61)
Not sure	13 (3.41)	17 (2.8)
Decline to answer	6 (1.57)	8 (1.32)
Missing	9	
Substance use over the last 30 days (check all that apply)		
Heroin	307 (80.57)	442 (71.75)
Opiates	153 (40.15)	194 (31.49)
Sedatives	115 (30.18)	125 (20.29)
Amphetamines	224 (58.79)	277 (44.97)
Cannabis	205 (53.80)	262 (42.53)
Missing	9	
Any substance use via injection in the past 6 months^a^		
Yes	313 (82.15)	437 (70.94)
No	68 (17.85)	179 (29.06)

^a^Polysubstance use was commonly observed among our sample. Substances may have been administered through multiple other routes as well

PrEP specific variables among the final analytic sample are presented in Table 2. Of the total sample (N = 997), a majority of participants (n = 770; 75.3%) reported not having heard much about PrEP. Few participants (n = 37; 4.0%) in the sample had used PrEP in the past with even fewer (n = 14; 1.4%) currently taking PrEP at the time of study participation. Almost one quarter (n = 222; 22.5%) of participants stated that they knew how to get PrEP if they wanted to start taking it. A larger percentage (n = 381; 38.2%) of participants indicated likelihood to accept LA (3-month) injectable PrEP.

**Table 2:** PrEP-Specific Variables among HIV negative participants (N = 997)

	N	%
How much have you heard about PrEP?		
A good amount	191	19.3
Not much	752	76
Decline to answer	46	4.6
Missing	8	
Have you ever taken PreP?		
Yes	37	3.7
No	899	91.1
Not sure	21	2.1
Decline to answer	30	3
Missing	10	
Are you currently taking PrEP?		
Yes	12	27.3
No	28	63.6
Decline to answer	4	9.1
If you wanted to start taking PrEP, would you know how to get it?		
Yes	222	22.5
No	563	57.2
Not sure	165	16.7
Decline to answer	35	3.5
Missing	12	
How likely would you be to use injectable PrEP every month?		
Likely	322	32.4
Not likely	671	67.6
Missing	4	
How likely would you be to use injectable PrEP every 3 months?		
Likely	381	38.2
Not likely	616	61.8
How likely would you be to use a lubricant that included PrEP?		
Likely	320	32.3
Not likely	672	67.7
Missing	5	
How likely would you be to use spermicide that included PrEP?		
Likely	290	29.8
Not likely	683	70.2
Missing	24	

80 participants among the 1077 HIV-negative participants did not respond to our outcome variable, “How likely would you be to use injectable PrEP every 3 months?”. Hence, the analytical sample comprises 997 participants

Bivariate logistic regression of demographic and other correlated predictors on the acceptability of LA injectable PrEP are presented in Table 3. Stepwise selection strategy was used by introducing race, study site, education, gender/sexual orientation into the multivariable logistic regression model (AIC = 1220.64). Injection drug use in the past six months was significantly associated with LA injectable PrEP acceptability, with the odds of acceptability being 1.885 (95% CI = 1.376, 2.582) being greater than those who did not inject drugs. Hispanic and Black participants (OR = 0.55, 95% CI = 0.395, 0.768; OR = 0.674, 95% CI = 0.48, 0.95, respectively) were statistically significantly less likely to accept LA injectable PrEP compared to White participants.

**Table 3:** Bivariate logistic regression of predictors of the likelihood of PrEP acceptability every 3 months among non-HIV-positive individuals in the Health Behaviors among Opioid Users study (N = 997)

	cOR	95% CI	*p*-value
Race/ethnicity (N = 962)			
White	Reference	–	–
Hispanic	0.550**	0.395, 0.768	0.0004
Black	0.674*	0.48, 0.946	0.0224
Other	0.655	0.386, 1.113	0.1176
Born in the US (N = 997)			
Yes	1.327	0.71, 2.48	0.377
No	Reference	–	–
Gender/sexual orientation (N = 979)			
Heterosexual men	0.531*	0.324, 0.869	0.0119
Heterosexual women	0.807	0.477, 1.363	0.421
Sexual minority men	Reference	–	–
Sexual minority women	0.915	0.515, 1.627	0.762
Relationship status (N = 981)			
Partnered	1.036	0.672, 1.598	0.87
Ever married	1.075	0.815, 1.1418	0.609
Never married	Reference	–	–
Education (N = 989)			
Up to High School	Reference	–	–
Some college/vocational education	1.761**	1.34, 2.316	< 0.0001
Higher education	1.782*	1.069, 2.971	0.0267
Employment status (N = 997)			
Unemployed and looking for work	Reference	–	–
Unemployed and not looking for work	1.003	0.683, 1.472	0.988
Out of workforce	1.012	0.719, 1.425	0.95
Employed	1.108	0.781, 1.57	0.57
Other	0.891	0.347, 2.287	0.81
Controlled environment in the past 6 months (N = 982)			
Yes, in the past 6 months	Reference	–	–
Not in the past 6 months	1.235	0.93, 1.641	0.145
Never held in a controlled environment	1.079	0.701, 1.662	0.729
Injectable substance use (N = 997)			
Yes	1.885**	1.376, 2.582	< 0.001
No	Reference	–	–
Location (N = 997)			
Atlanta	1.568*	1.113, 2.208	0.01
Las Vegas	1.375*	1.027, 1.84	0.032
Los Angeles	Reference	–	–
Insurance access (N = 997)			
Yes	0.855	0.634, 1.153	0.3
No	Reference	–	–

*cOR* crude odds ratio, *CI* confidence interval

^*^Indicates *p* < 0.05

^**^Indicates *p* < 0.01

Those who completed some college and post graduate education were statistically significantly more likely to accept LA injectable PrEP compared to those who had a high school education or less (cOR = 1.761, 95% CI = 1.34, 2.316; OR = 1.782, 95% CI = 1.069, 2.971, respectively). The odds of accepting LA injectable PrEP among heterosexual men was 0.53 (95% CI = 0.324, 0.869) times that of SMM; this association was statistically significant. While heterosexual women and sexual minority women were also less likely (cOR 0.807, CI 95% = 0.477, 1.363; cOR 0.915, 95% CI = 0.515, 1.627) to accept LA injectable PrEP compared to SMM, these associations were not statistically significant.

The odds of accepting LA injectable PrEP among participants who lived in Las Vegas were 1.37 times (95% CI = 1.027, 1.84) that of those who lived in Los Angeles. Similarly, the odds of accepting LA injectable PrEP among participants who lived in Atlanta were 1.57 times (95% CI = 1.376, 2.208) that of those who lived in Los Angeles. Injection drug use, race/ethnicity, gender identity/sexual orientation, educational attainment, and location were all statistically significantly and independently associated with the outcome variable.

In Table 4, we present the adjusted logistic regression model to evaluate the association between injection drug use and LA injectable PrEP acceptability among our sample. Case-wise deletion rate was less than 10% due to missing values for the explanatory variables. After controlling for education, study site, race/ethnicity, and gender/sexual orientation, injection drug use was significantly associated with the outcome, such that the odds of acceptability of LA injectable PrEP were 1.705 times greater among PWID compared to those who did not inject drugs (*p* < 0.03).

**Table 4:** Adjusted logistic regression predicting acceptability of PrEP every 3 months among non-HIV positive individuals in the Health Behaviors among opioid users study (N = 945)

Predictor	aOR	95%CI	*p*-value
Injection drug use^a^	1.705**	1.198, 2.427	0.003

*aOR* adjusted odds ratio, *CI* confidence interval

^a^The model was controlled for education, gender/sexual orientation, race and location

^*^Indicates *p* < 0.05

## Discussion

Findings from this project describe and highlight the acceptability of 3-month LA injectable PrEP among PWID in three urban settings. Our results are consistent with previously reported findings that similarly describe low awareness and knowledge of PrEP yet considerable acceptability of PrEP among this population with a novel focus on LA injectable PrEP [18–22]. Increasing the use of PrEP among PWID has recently been identified as a primary goal of a joint federal, state, and local response to the HIV epidemic across the U.S. after such preliminary research has shown PrEP to be efficacious within this population [30, 31]. Our conclusions highlight the need for additional education for PWID and improved access to PrEP and HIV prevention through addressing situational barriers (e.g., cost, insurance access, stable housing), as well as the importance of lessening barriers to PrEP within this population through effective and relevant policy-level change.

Of the limited research to date, findings indicate acceptability of PrEP among PWID alongside considerable efficacy in HIV prevention among different sub-populations of injection drug users [21, 22, 32, 33]. PWID in our sample were nearly twice as likely to accept 3-month injectable PrEP compared to non-PWID. Given the strong recommendation that PWID utilize PrEP to prevent HIV acquisition [34], ensuring access, acceptability, and utilization of PrEP among this population is critical [35, 36]. While interest in LA injectable PrEP was present within our sample, interest in other methods varied with considerably less interest. Lower acceptability of these products may be due to issues around access and transience within this community which create various barriers to following a strict PrEP regimen.

Similar to our findings, there have been other reports that identified a general lack of awareness and knowledge about PrEP among PWID [21, 22, 32, 33]. PrEP was initially primarily marketed directly towards SMM with little to no consideration of PrEP acceptability or appropriateness for other populations. Perceived stigma associated with PrEP use and a person’s sexuality or perceived sexual behaviors (e.g. multiple partners), has previously been reported among other populations (e.g., SMM), leaving possibility that stigma or hesitance to use PrEP due to perceived stigma could prevent use and longterm retention of an injectable PrEP regimen [37, 38]. However, injectable PrEP proves to be considerably more subtle as it does not require daily use or regular possession of medication. Additional research on the efficacy and usability of daily oral PrEP and injectable PrEP are being conducted with PWID [24].

While knowledge and awareness of PrEP remain low among PWID, high acceptability of PrEP has been reported. Research has shown that, of those aware of PrEP, over 60% were interested in taking it [27]. Our project also identified that of those aware of PrEP, high acceptability and interest in PrEP existed. Other studies have also presented that interest in PrEP alone is not enough, as PWID may perceive their own HIV risk profile as lower than it may be in reality, or that use of PrEP within this population may be intermittent or non-routine [33].

Within our project, we identified geographic differences in LA injectable PrEP acceptability, with Las Vegas and Atlanta having higher acceptability as compared to Los Angeles. While further research is needed, this may be due in part to the increased number of community-based organizations and syringe exchange facilities and resources available within Los Angeles County (compared to the other sites). In communities like Las Vegas and Atlanta, syringe exchange continues to be heavily stigmatized and prevented by local policies resulting in a limited number of resources to access [39, 40]. In constrast, Los Angeles is home to several harm reduction organizations, which increases access and potentially promotes greater awareness of PrEP among LA participants. Due to this, PWID in Los Angeles may have greater access to daily oral PrEP and HIV prevention resources than those in Las Vegas and Atlanta.

SSPs and other community-based organizations, like those involved in our project, offer a variety of resources and services to PWID, ranging from needle exchange to provision of condoms and STI testing, and could potentially incorporate PrEP information, screening, and provision. Recent research has identified that provision of PrEP through SSPs is a highly effective method to increase PrEP usage among this population [40–42]. PrEP delivery may be facilitated through partnerships with local clinics or other health-focused organizations to provide access to transient, low income, and potentially underinsured and homeless PWID. Fostering trust among PWID and healthcare providers can create additional opportunity for PrEP provision to PWID [35, 36]. Introduction of PrEP delivery and services at syringe exchange programs and other community-based organizations, where trust may be more easily fostered, will ensure the provision of holistic HIV prevention services for this population. [36] Where direct provision of PrEP and LA injectable PrEP may not be possible, provision of comprehensive PrEP education should be made a priority.

Individual community-based organizations and SSPs may be a direct access point to PrEP for PWID; however, policy level change is needed to adequately address the various barriers which exist and prevent PWID from regularly accessing PrEP. In 2019, the US Preventive Services Task Force issued a statement strongly recommending physicians to offer PrEP to persons at high risk of HIV acquisition, including PWID [43]. That same year, the *Ending the HIV Epidemic: A Plan for America* was introduced, which aims to end the HIV epidemic in the US by 2030 through targeted and strategic resource dissemination [44]. California also recently passed SB159 which stipulates that pharmacists are able to provide a 60-day supply of PrEP to anyone without a physician’s prescription if certain criteria are met [45]. California, like some other states, also passed legislation that provides funds to community-based organizations providing HIV prevention services, including PrEP [46]. In these settings, individuals may be able to access PrEP services, without attending traditional health services (e.g., clinic or hospital setting).

These recently passed policies indicate state-level support and movement for HIV prevention and removal of various barriers to PrEP. While states like California have made positive moves towards increasing HIV prevention and care among all at risk populations, others, focus more specifically on SMM populations in their prevention and treatment efforts, identifying a clear opportunity for further expansion of efforts to other at risk populations, including PWID. Further, considerations around cost and insurance coverage should also stay at the forefront when considering access to PrEP, especially as new, potentially more expensive, methods of prevention arise. In populations that are transient, much like our project sample, LA PrEP may be an effective HIV prevention method, but only if the population is able to access PrEP at reduced or no cost.

## Limitations

Our project was cross-sectional in design and thus is limited in understanding of variable associations. As our survey was self-administered; some possiblility of social desirability bias exists; however, staff provided participants ample space and time to complete the survey so as to avoid any sense of pressure or outward involvement from the staff person. As our sample is drawn from the three selected cities, our findings are not representative of other geographic regions, or PWID outside of SSPs. Our findings indicate that additional research in this area is needed to develop further understanding of the acceptability of LA injectable PrEP among PWID.

Currently, 2-month injectable PrEP, an additional form of PrEP which has been in development, seems to be moving through research processes and potential approvals. Our research does not provide evidence to support acceptability of 2-month injectable PrEP among IDU as our focus was on 3-month injectable PrEP. We also do not provide a direct comparison to daily oral PrEP as the focus of this project was on injectable PrEP. At the time of data collection, injectable PrEP was only emerging and very preliminary data had been conducted. However, our findings indicate higher acceptability of 3-month injectable PrEP, as compared to 1-month injectable PrEP highlighting a potential higher acceptance for longer-lasting injectable PrEP.

## Conclusions

Despite these limitations, our research provides important direction for future research on LA injectable PrEP with PWID. While injectable PrEP options are emerging, and efficacy of these methods within PWID are being studied, planning for and considerations around the provision of injectable PrEP to PWID populations is crucial to securing an end to the HIV epidemic. Utilizing SSPs as a vital access and intervention point to inform clients about LA injectable PrEP and distribute to clients will likely be an essential strategy to distribute LA injectable PrEP which may be an effective and efficient approach to reducing new HIV transmissions and overall HIV burden. Policy considerations suggest a focus on access to and retention on PrEP regimens of LA injectable PrEP. Lastly, SSP and other harm reduction organizations should make an effort to draft targeted messaging for PWID to encourage use of injectable PrEP and retention on a PrEP regimen. Through efforts to increase acceptance and regular use of LA injectable PrEP, public health strategies may be able to effectively lessen chances of future HIV outbreaks among PWID.

## Data Availability

The datasets used and/or analysed during the current study are de-identified and available from the corresponding author on reasonable request.
